# Deep Brain Stimulation for Alzheimer's Disease: Stimulation Parameters and Potential Mechanisms of Action

**DOI:** 10.3389/fnagi.2021.619543

**Published:** 2021-03-11

**Authors:** Yinpei Luo, Yuwei Sun, Xuelong Tian, Xiaolin Zheng, Xing Wang, Weina Li, Xiaoying Wu, Bin Shu, Wensheng Hou

**Affiliations:** ^1^Key Laboratory of Biorheological Science and Technology of Ministry of Education, Chongqing University, Chongqing, China; ^2^Chongqing Medical Electronics Engineering Technology Research Center, Chongqing University, Chongqing, China; ^3^Department of Neurosurgery, Southwest Hospital, Third Military Medical University (Army Medical University), Chongqing, China; ^4^Department of Rehabilitation Medicine, University-Town Hospital of Chongqing Medical University, Chongqing, China

**Keywords:** deep brain stimulation, Alzheimer's disease, therapeutic effect, stimulation parameter, action mechanism

## Abstract

Deep brain stimulation (DBS) is a neurosurgical technique that regulates neuron activity by using internal pulse generators to electrodes in specific target areas of the brain. As a blind treatment, DBS is widely used in the field of mental and neurological diseases, although its mechanism of action is still unclear. In the past 10 years, DBS has shown a certain positive effect in animal models and patients with Alzheimer's disease (AD), but there are also different results that may be related to the stimulation parameters of DBS. Based on this, determining the optimal stimulation parameters for DBS in AD and understanding its mechanism of action are essential to promote the clinical application of DBS in AD. This review aims to explore the therapeutic effect of DBS in AD, and to analyze its stimulation parameters and potential mechanism of action. The keywords “Deep brain stimulation” and “Alzheimer's Disease” were used for systematic searches in the literature databases of Web of Science and PubMed (from 1900 to September 29, 2020). All human clinical studies and animal studies were reported in English, including individual case studies and long-term follow-up studies, were included. These studies described the therapeutic effects of DBS in AD. The results included 16 human clinical studies and 14 animal studies, of which 28 studies clearly demonstrated the positive effect of DBS in AD. We analyzed the current stimulation parameters of DBS in AD from stimulation target, stimulation frequency, stimulation start time, stimulation duration, unilateral/bilateral treatment and current intensity, etc., and we also discussed its potential mechanism of action from multiple aspects, including regulating related neural networks, promoting nerve oscillation, reducing β-amyloid and tau levels, reducing neuroinflammation, regulating the cholinergic system, inducing the synthesis of nerve growth factor.

## Introduction

Alzheimer's disease (AD) is the most common type of dementia in the elderly. Its clinical manifestations are progressive cognitive decline and memory loss (Querfurth and LaFerla, 2010; Patterson, 2018). The prevalence and incidence of AD are increasing rapidly, and it is one of the major health crises facing the aging society of the 21st century (Alzheimer's Association, 2020). Clinically, only a small number of AD patients benefit from the temporary treatment effect of AD drugs. There are currently no viable medications to slow or reverse the progression of AD (Ihl et al., 2011; Arai et al., 2016, 2018; Cummings et al., 2020). The severity of AD and the limitations of drug therapy have spurred the development of research on non-drug therapies (Cummings et al., 2014; Aldehri et al., 2018). A series of physical therapy methods, including electrical stimulation and magnetic stimulation, are gradually being applied in the field of neurological diseases (Li et al., 2015; Temel and Jahanshahi, 2015; Zhou et al., 2018; Holczer et al., 2020).

Aldini first reported the use of electrical stimulation technology to improve the mood of melancholy patients in 1804, thus opening up a new field of electrical stimulation for the clinical treatment of mental illness (Aldini, 1804; Parent, 2004). Deep brain stimulation (DBS) is an invasive neuromodulation technique that involves brain stimulation and is one of the few neurosurgery methods that allows blinded research. The DBS device is mainly composed of stimulating electrodes in the brain, subcutaneous leads and pulse generators, and it directly changes brain activity in a controlled manner by using internal pulse generators to deliver electrical pulses to stimulation electrodes in specific target areas of the brain (Kringelbach et al., 2007; Lozano and Lipsman, 2013; Chen and Ponce, 2019). DBS is used for mental disorders, such as obsessive-compulsive disorder, epilepsy and depression (Zhou et al., 2018; Vazquez-Bourgon et al., 2019; Onate-Cadena et al., 2020), and for a variety of neurodegenerative diseases, such as idiopathic tremor, Parkinson's disease, and dystonia (Miocinovic et al., 2013; Chen and Ponce, 2019; Lee et al., 2019; McKinnon et al., 2019). Human clinical trials of DBS for AD began in 1984. Turnbull applied DBS to the left nucleus basalis of Meynert (NBM) in patients with a 4-years AD course but found no improvement in memory or cognition (Turnbull et al., 1985). Since then, research on treatment with DBS in AD has been vacant for more than two decades.

Research on the use of DBS in AD was restarted by a case study that used DBS to treat obese patients. The memory of a morbidly obese patient was enhanced after chronic hypothalamic/fornix DBS treatment. This enhancement may have occurred because DBS regulates marginal activity. That is, electrical activity in the medial temporal lobe is activated during DBS stimulation (Hamani et al., 2008). In the past 10 years, preliminary studies of DBS in AD have shown some positive effects of this treatment, including slowing cognitive decline and hippocampal atrophy and increasing cerebral glucose metabolism and brain connectivity in AD patients (Laxton et al., 2010; Sankar et al., 2015; Lozano et al., 2016; Aldehri et al., 2018), but there are also some controversial negative effects, such as AD patients <65 years of age have not shown good curative effects, and some patients have postoperative side effects (Lozano et al., 2016; McMullen et al., 2016; Leoutsakos et al., 2018). These discrepant results cannot be ignored. Differences in methodology, such as DBS parameters (stimulation target, frequency, stimulation start time, duration, unilateral/bilateral treatment, and stimulation current intensity), study design and sample size may explain these differences. The exact mechanism of action of DBS in AD is unknown. At present, there is little direct research on the mechanism of action of DBS in AD, and various hypotheses (changing the electrical activity of neurons, promoting neurogenesis and neurotransmitter release, etc.) have been proposed to explain its potential mechanism of action (Hescham et al., 2013a; Laxton and Lozano, 2013; Kuhn et al., 2015a; Aldehri et al., 2018; Jakobs et al., 2019; Yu et al., 2019).

There is increasing interest in exploring DBS as a treatment method for intervention in AD. A decision analysis model of DBS in AD patients shows that DBS is more effective and more cost-effective than standard treatment in the clinical treatment of AD (Mirsaeedi-Farahani et al., 2015). To effectively promote the application of DBS in the field of AD treatment, in this review, we systematically searched the literature published in the field of DBS in AD, and explored the effect of DBS stimulation parameters on the treatment effect for AD and the potential mechanism of action of DBS in the treatment of AD.

## Methods

### Literature Search and Selection Criteria

In the literature databases of Web of Science and PubMed (from 1900 to September 29, 2020), human clinical studies and animal studies on DBS in AD were systematically searched using the following terms, individually and combined in multiple search strategies: “Deep brain stimulation,” “DBS,” “Alzheimer's disease,” and “AD.” Literature inclusion criteria: the main purpose was to study the therapeutic effects of DBS in AD, including human clinical research and animal research; the language of the article was English only; individual case studies and long-term follow-up studies were not excluded; and duplicate studies were excluded.

### Data Abstraction

The selected articles were classified according to human clinical research and animal research to extract relevant data. The following data were extracted from human clinical studies: (1) study design; (2) basic information on the participants, including number, sex, average age, average Alzheimer's disease assessment scale-cognitive section (ADAS-Cog), average Mini-Mental State Examination (MMSE), and additional medications; (3) DBS data, including the DBS protocol and brain target; (4) follow-up; (5) main results, including the main outcome measures (average ADAS-Cog and average MMSE) and conclusion; and (6) adverse events. Various cognitive outcome measures have been used in human clinical studies, and some studies have evaluated multiple indicators. In this review, we chose the average ADAS-Cog and average MMSE as the main outcome measures. For studies that did not report averages and SDs, data were calculated from specific figures in the literature. The following data were extracted from animal studies: (1) basic information on the animals, including number, sex, age, animal type, grouping; (2) DBS data, including DBS protocol and brain target; (3) behavioral methods; (4) follow-up; and (5) main conclusion.

## Results

### Search Results

**Figure 1:**
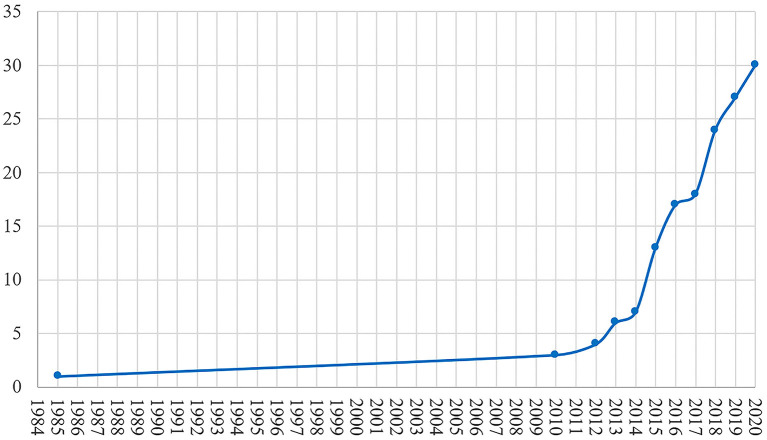
The cumulative growth of studies of DBS in AD.

This review included 30 studies on the treatment of DBS in AD. As shown in Figure 1, research on DBS in AD treatment was lacking for ~25 years after 1984 when DBS was applied in AD treatment. The time on the abscissa was the publication time of the article. It was not until 2010 that there was another paper on DBS in AD. During the 10-years period from 2010 to 2020, DBS in AD papers were published every year.

### Human Clinical Studies

#### DBS Implementation

DBS is implemented in the human body and its components are surgically implanted. As mentioned earlier (Laxton et al., 2010; Mao et al., 2018), after local anesthesia is applied, the stereotaxic frame is installed on the patient's head. The position of the DBS target is ascertained by imaging. Subsequently, a drill is used to make bilateral holes in the skull, and the stimulation electrodes are implanted three-dimensionally. After the electrodes are placed, the internal pulse generator is implanted into the subclavian or subcutaneous area of the chest or abdomen under general anesthesia. The lead is implanted subcutaneously and connected to the stimulation electrodes and internal pulse generator through the head, neck, and chest. The positioning of the electrodes must be verified again by imaging after the operation.

#### Therapeutic Effects

Table 1 lists the detailed characteristics of 16 human clinical studies of DBS in AD patients. Turnbull et al. started the field of DBS in the treatment for AD in 1984 (Turnbull et al., 1985). A study by Laxton et al. (2010) showed that after DBS treatment, the memory of AD patients improved, the rate of cognitive decline decreased, and cerebral glucose metabolism increased (Laxton et al., 2010; Smith et al., 2012). Subsequently, human clinical studies of DBS in AD continued. Fontaine et al. reported that a patient with AD showed stable memory and increased metabolism in the medial temporal lobe after 1 year of DBS (Fontaine et al., 2013). Other studies have also shown that the nutritional status of AD patients remains stable, and the rate of hippocampal atrophy slows down after 1 year of DBS (Noreik et al., 2015; Sankar et al., 2015). Kuhn et al. applied DBS to 6 AD patients, and 1 year later, the ADAS-Cog scores of four participants increased or remained stable, but the scores of two participants deteriorated (Kuhn et al., 2015a). Kuhn et al. also performed DBS on two other AD patients who were 5 years younger. One AD patient had a stable ADAS-Cog score and an increase in MMSE score after 28 months. Another case showed overall improvement in the first year of treatment, but the ADAS-Cog score increased by seven points after 26 months (Kuhn et al., 2015b). The long-term follow-up results of all eight patients at 24 months showed that the ADAS-cog of young patients with mild AD remained relatively stable during the 24-months follow-up period, while the ADAS-cog of AD patients with high baseline increased (Hardenacke et al., 2016). In the two youngest patients among the eight, the repetitive inhibition of sensory gating improved (Durschmid et al., 2020). Therefore, they believed that DBS performed in the early and young ages of AD may have a beneficial effect on the progression of the disease and cognitive function. Baldermann et al. also showed that AD patients with less atrophy can benefit from DBS (Baldermann et al., 2018). However, the study by Lozano et al. showed different results. Lozano et al. observed an increase in cerebral glucose metabolism in 21 AD patients after 1 year of DBS, considered DBS to be safe, and proposed that DBS may benefit AD patients aged 65 years and older, while those under 65 years old may show a worsening condition (Lozano et al., 2016). Leoutsakos et al. conducted a second-year follow-up of 42 participants in the study, confirming that DBS benefits older patients with AD (Leoutsakos et al., 2018). Furthermore, McMullen et al. reported that an AD patient implanted with bilateral fornix DBS experienced asymptomatic bilateral cerebral encephalomalacia (McMullen et al., 2016). Among the current existing studies, only two studies have shown no therapeutic effect of DBS in AD (Turnbull et al., 1985; McMullen et al., 2016), and there were a few AD patients with poor performance in other studies (Lozano et al., 2016; Leoutsakos et al., 2018; Mao et al., 2018). The small sample size (case report) (Turnbull et al., 1985; McMullen et al., 2016), side effects of DBS surgery (Ponce et al., 2016), and improper use of quetiapine by patients (Lozano et al., 2016; Leoutsakos et al., 2018; Mao et al., 2018) may account for these differences, but we cannot rule out the possibility that it is due to other methodological differences. However, among most AD patients receiving DBS, DBS is a promising intervention for AD.

**Table 1:** The human clinical study of DBS in AD.

**Study (first author, year)**	**Study design**	**Baseline**						**DBS protocol**	**Brain target**	**Follow up**	**Main results**		
											**Main outcome measures**		**Conclusion**
		***N***	**Sex (male/female)**	**Average age (y)**	**Average ADAS-Cog**	**Average MMSE**	**Medication (add-on-therapy)**				**Average ADAS-Cog**	**Average MMSE**	
Turnbull et al. (1985)	-	1	1	74	-	-	-	3 V, 50 Hz, 210 ms, on for 15 s and off for 12 min, 9 m	L-NBM	Followed DBS	-	-	There was no significant clinically effect.
Laxton et al. (2010)	-	6	4/2	60.7 ± 5.5	-	22.3 ± 4.1	Cholinesterase inhibitors (least 6 m)	3.0~3.5 V, 130 Hz, 90 μs,12 m	B-fornix	1 m, 6 m, 12 m	-	-	DBS drove neural activity in the memory circuit, activated the brain's default mode network and could improve the cognitive ability of some patients with mild AD.
Smith et al. (2012)	-	5	4/1	62.6 ± 4.2	19.2 ± 7.2	22.2 ± 5.1	cholinesterase inhibitors (least 6 m)	3.0~3.5 V, 130 Hz, 90 μs,12 m	B-fornix	1 m, 12 m	1 m, 21.6 ± 9.2; 12 m, 23.9 ± 13.7	-	DBS increased cerebral glucose metabolism of patients with mild, probable AD in 2 orthogonal networks.
Fontaine et al. (2013)	-	1	0/1	71	3m before DBS, 12.25; 7d before DBS, 9.	3m before DBS, 23; 7d before DBS, 29.	-	2.5V, 130Hz, 210 ms, 12 m	B-fornix	3 m, 6 m, 12 m	3 m, 9.41; 6 m, 10; 12 m, 9.91	3 m, 25; 6 m, 26; 12 m, 24	During 1 year of DBS, the memory scores of an AD patient remained stable, and its metabolism increased in the mesial temporal lobes.
Kuhn et al. (2015a)	double blind, crossover design	6	2/4	69.5 ± 7.7	20.2 ± 6.0	20.3 ± 2.5	Acetylcholinesterase medication	Randomized sham-controlled: −0, −8, +case, 20 Hz, 90 μs and 2.5 V, on for 2 w and off for 2 w; followed by continuous individualized stimulation: 2.0-4.5 V, 10-20 Hz and 90-150 μs	B-NBM	6 w, 12 w, 26 w, 52 w	6 w, 19.0 ± 10.0; 12 w, 20.0 ± 8.0; 26 w, 20.0 ± 9.0; 52 w, 23.2 ± 13.0	6 w, 19.3 ± 4.0; 12 w, 18.8 ± 3.1; 26 w, 19.2 ± 5.3; 52 w, 18.7 ± 6.7	ADAS-cog scores worsened by an average of three points after 1 year of stimulation, and the mean MMSE score remained almost stable. Bilateral low-frequency DBS of the NBM in AD patients is technically feasible and tolerable.
Kuhn et al. (2015b)	Double blind	2	-	64 ± 2.12	10 ± 0.71	22 ± 2.12	-	Continuous individualized stimulation: 20 Hz	B-NBM	12 m, 26 m	12 m, 7.5 ± 0.35; 24 m, 13.5 ± 1.77	12 m, 26 ± 1.41; 24 m, 22 ± 0.71	NBM DBS performed may have a favorable impact on disease progression at the early stage of AD.
Noreik et al. (2015)	Proof-of-concept	6	2/4	69.2 ± 7.5	-	-	-	2.0-4.5 V, 10-20 Hz and 90-150 μs, 2 w ON, 2 w OFF or vice versa, followed by continuous stimulation	B-NBM	1 year	-	increased by 3 points (min −9, max 19)	AD patients treated with NBM DBS demonstrated a mainly stable nutritional status within 1 year.
Sankar et al. (2015)	Controlled	31	DBS: 4/2 Control: 16/9	DBS: 60.7 ± 6.1; Sham, 63.9 ± 4.4	DBS, 19.1 ± 6.4; Sham, 18.7 ± 7.0	DBS, 22.3 ± 4.5; Sham, 23.6 ± 1.8	acetylcholinesterase medication (least 6 m)	3.0 V, 130 Hz, 90 μs, 12 m	B-fornix	1 year	DBS: 23.33 ± 12.3; Sham, 23.8 ± 10.6	DBS: 21.5 ± 6.2;Sham, 19.7 ± 4.8	The mean hippocampal atrophy of AD patients after DBS became slower.
Lozano et al. (2016)	Randomized, double blind, controlled, multi-center	42	DBS,11/21 (≥65, 15); Sham, 12/21 (≥65, 15)	DBS, 68.5 ± 7.7; sham, 67.8 ± 8.1	DBS, 28.6 ± 3.9; sham, 27.1 ± 3.8	-	cholinesterase inhibitor (least 2 m)	3.0~3.5 V, 130 Hz, 90 μs, 12 m	B-fornix	1 m, 6 m, 9 m, 12 m	1 m, DBS, 28.0 ± 7.7, sham, 28.9 ± 7.4; Difference of DBS and sham: <65, 12m, 10.3 ± 6.1; ≥65, 9 m, 4.5 ± 2.0, 12 m, 4.1 ± 2.6.	-	DBS may have a positive effect on AD patients ≥65 years old, but may have an adverse effect on patients under 65 years old.
Hardenacke et al. (2016)	-	8	-	-	-	-	-	-	B-NBM	12 m, 18 m, 24 m	-	-	NBM DBS performed may have a favorable impact on disease progression at the early stage of AD.
McMullen et al. (2016)	Double-blind, randomized	1	1	48	19	-	-	-	B-fornix	3 m	22	-	A patient with AD who experienced fornix DBS developed bilateral encephalomalacia.
Baldermann et al. (2018)	-	10	5/5	66.9 ± 4.3	9.3 ± 6.5	18.3 ± 3.8	-	2.0-4.2 V, 5-20 Hz, 60-150 μs	B-NBM	6 m, 12 m	6 m, 10.9 ± 8.1; 12 m, 11.6 ± 11.2	6 m, 17.9 ± 5.8; 12 m, 20.1 ± 6.6	AD patients with less advanced atrophy may profit from NBM DBS.
Scharre et al. (2018)	-	3	-	63 ± 5.31	30.33 ± 3.66	22.67 ± 0.72	-	continuous stimulation for at least 18 m.	B- VC/VS	27 m, 24m, 21 m	-	-	DBS of the VC/VS was well-tolerated and the extent of CDR-SB decline in AD patients with VC/VS DBS was reduced.
Leoutsakos et al. (2018)	Double blind, controlled	42	DBS,11/21 (≥65, 15); Sham, 12/21 (≥65, 15)	-	-	-	Cholinesterase inhibitor (least 2 m)	Sham, DBS in the second year.	B-fornix	3 m, 6 m, 9 m, 12 m, 18 m, 24 m	DBS, first year, 7.83 ± 1.86, the second year, 5.60 ± 1.85; sham, first year, 8.33 ± 1.82, the second year, 6.16 ± 1.97.	-	Fornix DBS was safe and may be beneficial for AD patients ≥65 years of age.
Mao et al. (2018)	-	5	2/3	59 ± 1.79	-	2.4 ± 1.15	Cholinesterase inhibitor, Chinese medication. (least 6 m)	130 Hz, 90 ms, 1–5 V	B-fornix	1.5 m, 3 m	-	3 ± 1.33	Fornix DBS could improve partial improvement in performance of patients with severe AD, including cognitive performance, mental state and social performance.
Durschmid et al. (2020)	-	2	-	62 ± 0.71	-	-	-	1 V, 20 Hz	B-NBM	-	-	-	NBM DBS has a positive impact on sensory gating into memory.

*AD, Alzheimer's Disease; ADAS-Cog, Alzheimer's disease assessment scale-cognitive section; B, bilateral; CDR-SB, clinical dementia rating–sum of boxes; DBS, deep brain stimulation; L, left; m, mouth; min, minute; MMSE, mini-mental state examination; N, number; NBM, nucleus basalis of Meynert; Sham, sham stimulation; R-, right; VC/VS, ventral capsule/ventral striatum; w, week; y, year*.

### Animal Studies

#### DBS Implementation

For DBS in AD animal models, the stimulating electrodes are implanted, and the stimulator is connected externally. A single study implanted the stimulators subcutaneously on the backs of mice (Huang et al., 2019). Each mouse is anesthetized and fixed on a stereotaxic device; the scalp is removed, exposing the skull. An electric drill is used gently drill small holes at the target points on the skull. Then, the stimulating electrode is implanted at the target position and fixed to the skull with adhesive material. In some studies, a small number of screws are implanted in the skull to fix the electrodes (Hescham et al., 2013b; Chen et al., 2014; Zhang et al., 2015; Tsai et al., 2020). X-ray imaging or tissue staining is used to ensure that the electrode is implanted in the target position (Lee et al., 2016; Mann et al., 2018; Huang et al., 2019).

#### Therapeutic Effects

In addition to human clinical research on DBS in AD, animal research on DBS in AD is also being carried out simultaneously. Table 2 lists the detailed characteristics of 14 animal studies of DBS in AD animal models. Compared with human clinical studies, the behavioral, physiological and biochemical changes of AD model mice established by transgenic AD model or drug injection after DBS are mostly positive in the animal studies. This review later used these animal studies, combined with human clinical studies, to explore the effects of DBS stimulation parameters on the efficacy of AD treatment and the potential mechanism of action of DBS in AD.

**Table 2:** The animal study of DBS in AD.

**Study (first author, year)**	**Base**					**DBS protocol**	**Brain target**	**Behavior methods**	**Follow up**	**Main conclusion**
	***N***	**Sex (male/female)**	**Age**	**Animal**	**Group**					
Arrieta-Cruz et al. (2010)	16–20	male	8 w	TgCRND8, WT (B6C3F1)	WT, AD-DBS	Eight trains of HFS; each train of 25 Hz, 1 s duration, 300 μA, 10 s inter-train interval, 3 d	B-MTN, [AP: −1.1, ML: 0.3, DV: 2.5–3.3]	NOR	After DBS	DBS could enhance short-term memory in the CA1 region of hippocampus in TgCRND8 mice.
Hescham et al. (2013b)	21	21/0	-	An injection of scopolamine to SD rats	AD (*n* = 11), AD-DBS (*n* = 10)	50 μA, 100 μA, 200 μA, 10 Hz, 100 Hz, 100 μs, 2 min/time	B-fornix, [AP −1.88, ML 1.3, DV 8.2]	NLR, OF	After DBS	Fornix DBS reversed the memory of rats received scopolamine and it was not sensitive to stimulation frequency, but rather to current levels.
Chen et al. (2014)	32	32/0	-	An injection of Aß_1−42_ and Aß_1−40_ to SD rats	SD-PBS (*n =* 8), AD (*n* = 8), AD-ANT (*n* = 8), AD-DBS (*n* = 8)	130 Hz, 60 μs, 1.5 V	B-ANT, [AP: −2.0, ML: ± 1.8, DV: −4.7]	MWM	After DBS	Bilateral ANT HFS could improve the performance of AD rats in MWM.
Zhang et al. (2015)	48	48/0	6 w	An injection of Aß_1−42_ to SD rats	ANT DBS (*n =* 8), EC DBS (*n =* 8), fornix DBS (*n =* 8), ANT-sham (*n =* 8), EC-sham (*n =* 8), fornix-sham (*n =* 8)	500 μA, 130 Hz, 90 μs, 24 h	B-ANT, [AP: −1.6, ML: 1.5, DV: −5.2]; B-EC, [AP: −7.0, ML: 5.4, DV: −8.2]; B-fornix, [AP: −1.9, ML: 1.3, DV: −8.2]	MWM, NOR, OF	4 w after DBS.	EC and fornix DBS could enhance hippocampus-independent recognition memory, and facilitated hippocampus-dependent spatial memory more prominently than ANT DBS.
Hescham et al. (2015)	63	-	-	An injection of scopolamine to SD rats	Sham (*n =* 11), CA1 DBS (*n =* 10), MT DBS (*n =* 13), ANT DBS (*n =* 14), EC DBS (*n =* 15)	50 μA, 100 μA, 200 μA, 10 Hz, 100 Hz, 100 μs, 24 h	B-CA1, [AP: −3.6, ML: 1.8, DV: −2.6]; B-MT, [AP: −1.8, ML: 1, DV: −6.2]; B-ANT, [AP: −1.6, ML: 1.5, DV: −5.2]; B-EC, [AP: −6.7, ML: 4, DV: −8].	NLR, OF, EZM	After DBS	CA1, EC, and fornix DBS could able to restore spatial memory-related functions and CA1 DBS increased neural activity in the anterior cingulate gyrus.
Lee et al. (2016)	25	25/0	6 w	An injection of 192 IgG-saporin to SD rats	SD (*n =* 6), AD (*n =* 7), AD-NS (*n =* 7), AD-DBS (*n =* 5)	120 Hz, 90 μs, 1 V, 1 h/d, 7 d	R-NBM, [AP: −1.32, ML: +2.8, DV: −7.4]	MWM	After DBS	NBM DBS improved spatial memory performance of SD rats injected 192 IgG-saporin in the MWM.
Xia et al. (2017)	98	49/49	6 w, 6 m	TgCRND8, WT (C57BL/6NTac)	WT-NS (*n =* 50), AD-NS (*n =* 19), AD-DBS (*n =* 29)	130 Hz, 90 μs, 1 h, square wave	B-EC, [AP: −4.0, ML: ± 3.25, DV: −5.1]	Contextual fear testing; Tone fear testing; MWM	1, 3, and 6 w for behavior experiments after DBS.	EC DBS rescued subsequent deficits in context fear memory, reversed spatial learning deficits in Tg mice, and reduced plaque load in young mice. But EC DBS did not produce a detectable decrease in plaque load in either the dorsal hippocampus or cortex in old mice.
Akwa et al. (2018)	20	20/0	4 m	3 × Tg, WT (C57BL/6/129SVJ)	WT-NS (*n =* 5), WT-DBS (*n =* 5), AD-NS (*n =* 5), AD-DBS (*n =* 5)	50 μA, 130 Hz, 90 μs, 7 h/d, weekends off, 25 d	B-EC, [AP: −4.0, ML: ± 3.0, DV: −5.1]	-	-	Chronic DBS in 3 × Tg mice resulted in reduced levels of Tau oligomers, and increased levels of synaptophysin.
Mann et al. (2018)	38	38/0	4 m	3 × Tg, WT (129SV/C57BL6)	WT-Cont (*n =* 5), AD-Cont (*n =* 11), AD-NS (*n =* 10), AD-DBS (*n =* 12)	50 μA, 130 Hz, 90 μs, 7 h/d, weekends off, 25 d	B-EC, [AP: −4.0, ML: 3.0, DV:5.1]	MWM, NLR, NOR	5 m for MWM, 6.5 m for NPR and NOR; 7 m for euthanize.	Chronic EC DBS improved both memory and AD specific pathological markers of AD mice.
Leplus et al. (2019)	22	-	18 m	TgF344-AD, WT F344	WT (*n =* 6), WT-DBS (*n =* 6), AD (*n =* 4), AD-DBS (*n =* 6)	130 Hz, 80 μs, 100 μA, unipolar, 42 d	B-fornix, [AP: −0.6, ML: ± 0.75, DV: −5.8]	-	After DBS	Chronic DBS decreased amyloidosis, inflammatory responses, and neuronal loss in both cortex and hippocampus in AD rats.
Huang et al. (2019)	192	192/0	4 m, 6 m, 9 m, 12 m	APP/PS1 (HuAPP695swe, PSEN1-dE9), WT (C57BL/6)	Frequencies: control, sham, 10 Hz, 50 Hz, 100 Hz,130 Hz; Starting times: control, sham, 4 m, 6 m, 9 m, 12 m; Durations: control, sham, 7, 14, 21, 28 days; APP/PS1: control, sham, DBS; WT: control, DBS; The optimized parameters of DBS: control, sham, DBS, U0126, DBS+U0126, LY294002, DBS+LY294002	10 Hz, 50 Hz, 100 Hz,130 Hz, biphasic stimulus pulse wave, 7, 14, 21, 28 days	L-NBM, [AP: −0.7; ML: 1.75; DV: 4.0]	MWM	After DBS or 30 d after DBS or mice at 13 m	NBM DBS starting from 4 months of age for 21 days at 100 Hz had therapeutic effects on APP/PS1 mice through activating phosphatidylinositol 3′-kinase (PI3K)/Akt pathway and inhibiting ERK1/2 pathway.
Gallino et al. (2019)	50	AD-DBS, 9/8; Sham-DBS, 8/9; AD, 8/8.	2 m	3 × Tg	AD-DBS (*n =* 17), Sham-DBS (*n =* 17), AD (*n =* 16)	100 μA, 100 Hz, 100 μs, 1 h	B-fornix, [AP: 0; ML: 0.75; DV: 3.0]	MWM	MWM weekly and structural MRI in 3 d before and 3 d after DBS with a 6 w follow-up.	Acute DBS could improve learning and long-term memory of 3 × Tg mice in a delayed, sex specific, and transient manner.
Koulousakis et al. (2020)	12	12	18 m	TgF344-AD	AD-DBS	200 μA, 100 μs, 60 Hz for intermittent DBS (duty cycle: 20 s ON and 40 s OFF. 20 Hz, 120 μs for continuous DBS.	B-NBM, [AP: −1.44; ML: ± 2.88; DV: 7.4]. Intermittent, unilaterally or bilaterally; continuous, bilaterally.	OF, NOR, MBM	Before and after DBS.	Bilateral intermittent NBM DBS allowed aged TgF344-AD rats to perform better and maintain their performance longer in a spatial memory task.
Tsai et al. (2020)	37	37/0	-	An injection of Aß_1−42_ and Aß_1−40_ Wistar rats	Cont (*n =* 8), AD (*n =* 12), AD-sham (*n =* 8), AD-DBS (*n =* 9)	0.5 mA, 60 μs, 100 Hz, 30 min	R-ILN, [AP: −2.8, ML: 1.25, DV: −5.5]	MWM	After DBS	A single rostral ILN DBS could rescue spatial learning and memory deficits, and significantly reversed PSD-95 expression reductions and preserved dendritic spine densities in the mPFC and hippocampal region of Aβ-infused rats.

*Aβ, β-amyloid; AD, Alzheimer's Disease; ADAS-Cog, Alzheimer's disease assessment scale-cognitive section; ANT, anterior nucleus of thalamus; APP/PS1, amyloid-β precursor protein/presenilin1; B, bilateral; EC, entorhinal cortex; EZM, elevated zero maze; Cont, control; DBS, deep brain stimulation; DG, dentate gyrus; HFS, high frequency stimulation; ILN, intralaminar thalamic nucleus; L, left; m, mouth; MBM, modified barnes maze; min, Minute; mPFC, medial prefrontal cortex; MT, mammillothalamic tract; MTN, midline thalamic nuclei; MWM, morris water maze; N, number; NBM, nucleus basalis of Meynert; NOR, novel object recognition; NLR, novel location recognition; NS, non-stimulation; OF, open field test; SD, sprague dawley; Sham, sham stimulation; 3xTg, toronto triple transgenic; R, right; w, week; y, year; WT, wild type*.

### Adverse Events

DBS is an invasive brain stimulation technique that carries the risk of major surgery (Doshi, 2011). The main surgical complications include bleeding, infection, and hardware failure (Ponce et al., 2016; Barrett, 2017). The adverse events related to DBS that occur in AD are manifested in human clinical studies which are rarely found in animal studies. Table 3 lists the adverse events of the human clinical study of DBS in AD. At higher voltage intensity, AD patients felt inner restlessness, warmth, flushing, sweating, increased heart rate and blood pressure, and other adverse reactions. However, after reducing the voltage intensity, AD patients had almost no adverse effects (Laxton et al., 2010; Kuhn et al., 2015a). Other minor adverse reactions, including headache, diarrhea, vomiting, and paresthesias, were almost transient and had no sequelae (Laxton et al., 2010; Kuhn et al., 2015a; Leoutsakos et al., 2018; Scharre et al., 2018). Serious adverse events, including internal pulse generato infections, skin infections, inaccurate device location, hematoma, syncope, epilepsy, etc., could almost be resolved (Ponce et al., 2016). Leoutsakos et al. conducted the largest-scale study on the safety of DBS in AD patients, which involved 42 patients with AD (Lozano et al., 2016; Ponce et al., 2016; Leoutsakos et al., 2018). Within 90 days after receiving DBS surgery, 26 patients experienced 64 non-serious adverse events related to DBS surgery, of which 5 patients experienced 7 serious adverse events. For these 64 non-serious adverse events, 57 occurred within 30 days after surgery. In the second year after receiving DBS surgery, 24 patients reported 86 non-serious adverse events, and eight patients reported 15 serious adverse events. Two statistical results showed that the shorter the time after the completion of the operation is, the greater the possibility of reported adverse events in AD patients. The occurrence of adverse events gradually decreased over time, and there seemed to be no long-term complications. The safety of adverse events was as expected. However, the case report by McMullen et al. showed that DBS has brought long-term adverse effects (McMullen et al., 2016). This might be related to both the physical state of the patients and the experience of the surgeon. Although this was only a case study, researchers should pay attention to it. In general, AD patients tolerate DBS well under the appropriate DBS parameters, which proves that DBS is a relatively safe technique for AD patients.

**Table 3:** Adverse events of the human clinical study of DBS in AD.

**Study (first author, year)**	**Adverse events**
Turnbull et al. (1985)	Did not cause epilepsy or any other untoward reactions in a personal case.
Laxton et al. (2010)	When the voltage intensity of DBS was between 6 and 8 V, AD patients would experience adverse reactions, such as warmth, flushing, sweating, increased heart rate and blood pressure. When the stimulation intensity was reduced by 50%, there was almost no adverse effect.
Smith et al. (2012)	Not involved.
Fontaine et al. (2013)	An AD patient had no complications after DBS 1 year and was fully tolerant to stimulation, except a discrete increase of irritability.
Kuhn et al. (2015a)	The DBS device was malfunctioning; an AD patient felt inner restlessness at a stimulation intensity >5 V. There were no other adverse events.
Kuhn et al. (2015b)	Both patients were well-tolerated by DBS. There were no adverse events.
Noreik et al. (2015)	Not involved.
Sankar et al. (2015)	Not involved.
Lozano et al. (2016)	Three patients had 4 adverse events, including 1 internal pulse generato infection, 1 DBS positioning error, and 2 postoperative nausea (1 patient). No neurosurgical and cognitive adverse reactions.
Hardenacke et al. (2016)	Not involved.
McMullen et al. (2016)	At 3 months after surgery, the case patient was anxious and complained that “left side of brain is asleep,” and cystic encephalomalacia appeared in the frontal lobe; at 1 year after surgery, minimal encephalomalacia appeared on the bilateral lead.
Baldermann et al. (2018)	Not involved.
Scharre et al. (2018)	Short-term side effects were resolved without sequela, including hot flashes, increased heart rate/palpitations, flushing, paresthesias, muscle twitching, non-specific discomfort, fatigue, and neuropsychiatric symptoms, mild pain at implantable pulse generator site, headache at incision site, transient visual neglect following surgery, diarrhea, vomiting, rash, rhinitis, arthralgia, fall, hematoma, and depression.
Leoutsakos et al. (2018)	Twenty-four patients experienced non-serious adverse events, common ones including neurological (including falls, headache, and muscle spasms), genitourinary (including urinary tract infections, urgency, and incontinence), and pulmonary (including upper respiratory infections and dyspnea). Seven patients had syncope and/or falls; two patients had altered mental status; two patients were involved in seizures or possible seizure; one patient was involved in agitation in a delayed; three patients were involved in a skin infection, suspected aortic valve endocarditis, and rigidity.
Mao et al. (2018)	No serious neurological adverse events occurred.
Durschmid et al. (2020)	Not involved.

*AD, Alzheimer's Disease; DBS, deep brain stimulation*.

## DBS Parameters

### Stimulation Targets

**Figure 2:**
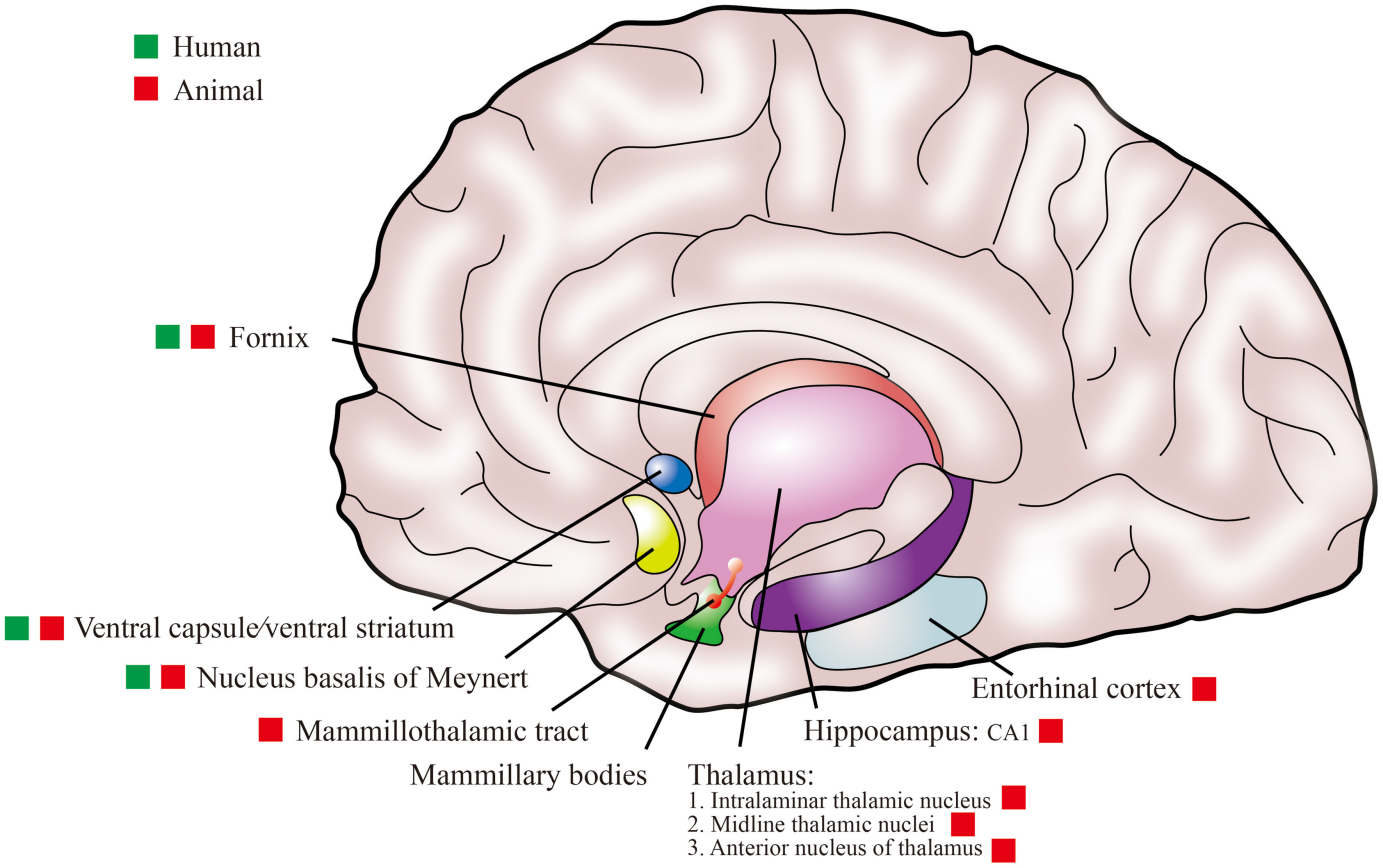
The schematic diagram of the stimulation targets for DBS in AD.

To date, the stimulation targets for DBS in the treatment of AD patients in human clinical studies have involved the fornix, NBM, and ventral capsule/ventral striatum (VC/VS) (Table 1). Relevant animal research also involved multiple stimulation targets, including the intralaminar thalamic nucleus (ILN), midline thalamic nuclei (MTN), mammillothalamic tract (MT), anterior nucleus of thalamus (ANT), entorhinal cortex (EC), and CA1 (Table 2). The selection of these stimulation targets for DBS is mainly based on the neural network of the brain. Among them, VC/VS is related to the frontal lobe neural network (Price and Drevets, 2010); ILN and MTN are important components forming cortico-thalamo-cortical pathways (Saalmann, 2014); NBM participates in the base forebrain cholinergic circuit; and the fornix, MT, ANT, EC, and CA1 (hippocampus) are nodes in the Papez circuit (Yu et al., 2019). Figure 2 shows a schematic representation of these targets in the brain. AD is also considered to be a disease of the neural circuit, as neurons and neural circuits associated with cognitive function are damaged, and the Papez circuit is degraded (Lv et al., 2018). The Papez circuit is the main pathway of the limbic system and plays a vital role in the formation and storage of memory. Understanding the effects of these stimulation targets can help to select the best DBS stimulation target for AD.

#### VC/VS

DBS regulates the frontal lobe network of the brain that is involved in cognition and behavior. The ventral striatum, nucleus accumbens, and anterior limb of the internal capsule sit at the base of the frontal lobes. White matter fibers of the frontal lobe and the ventral capsule connect the dorsomedial and orbital prefrontal cortices to the ventral striatum (Price and Drevets, 2010). These neural networks have shown degeneration in AD (Lehericy et al., 1989). VC/VS DBS can affect related behavioral disorders in patients with mental disorders such as obsessive-compulsive disorder and depression (Greenberg et al., 2010; Dougherty et al., 2015). In a non-random phase I experiment, Scharre et al. first used the VC/VS of the frontal lobe network as the stimulation target for DBS in AD patients (Scharre et al., 2018). Fluorodeoxyglucose-positron emission tomography metabolism increased, and clinical dementia rating–sum of boxes (CDR-SB) performance decreased in AD patients receiving DBS. By targeting the VC/VS, DBS may adjust the frontal lobe network and affect the executive function of AD patients. This is the first report to show improved behavioral and executive defects without tracking memory targets in the treatment of AD by DBS. It provides new target options for DBS treatment in AD. This study also shows that chronic DBS with the VC/VS as a target is well-tolerated.

#### ILN

The rostral part of the ILN is connected to the medial prefrontal cortex (mPFC) and is considered to be a key component of cognitive function (Mair and Hembrook, 2008; Saalmann, 2014). Although the function of the ILN in cognitive function has been proven, the effect of ILN DBS on AD-related cognitive dysfunction has not been extensively studied. Only one related study has shown that unilateral ILN DBS treatment can improve spatial learning and memory deficits in AD rat models injected with β-amyloid (Aβ), reduce the expression of postsynaptic density protein 95 (PSD-95) in the mPFC and hippocampus, and maintain dendritic spine density (Tsai et al., 2020).

#### MTN

The MTN connects to the medial prefrontal cortex, medial temporal lobe and hippocampus. The midline structure of thalamus is involved in memory related functions (Saalmann, 2014). An study of high-frequency MTN DBS showed that the time that TgCRND8 mice explored new objects in the object recognition task was significantly longer than that of wild type mice, and the expression of FosB protein in the hippocampus was significantly upregulated (Arrieta-Cruz et al., 2010). There is currently no other research on MTN DBS in AD.

#### NBM

Acetylcholine (ACh) is essential for cognitive function and memory processing, and it is mainly derived from cholinergic neurons in the NBM (Carlsen et al., 1985). This region is mainly located below the anterior commissure and globus pallidus and on the anterior lateral part of the hypothalamus (Hedreen et al., 1984). The NBM sends a wide range of cholinergic nerve projections that dominate the cortex and hippocampus, and it represents an important cholinergic pathway in functional networks that subserve cognition and memory (Gratwicke et al., 2013). NBM damage can lead to a decrease in cholinergic transmission and degenerative changes in mossy fibers and the dentate gyrus of the hippocampus (Bartus et al., 1982; Amenta et al., 1991). The first evidence neural network dysfunction in AD is the loss of NBM cholinergic neurons in the brain, which is evident on postmortem examination (Whitehouse et al., 1981). Turnbull et al. reported for the first time the impact of NBM DBS on AD patients, although the report found no evidence of clinical improvement (Turnbull et al., 1985). Since then, chronic NBM DBS has shown good efficacy in AD. NBM DBS performed in early AD may have beneficial effects on AD progression and cognitive function (Kuhn et al., 2015b; Hardenacke et al., 2016). AD patients receiving NBM DBS showed a substantially stabilized nutritional status within 1 year (Noreik et al., 2015). NBM DBS can also improve the spatial learning and memory of AD model mice; regulate the gamma-aminobutyric acid (GABA), glutamate system, and cholinergic systems; reduce the abundance of amyloid protein; and exert neuroprotective effects (Lee et al., 2016; Huang et al., 2019; Koulousakis et al., 2020).

#### Fornix

As part of the Papez circuit, the fornix is the main inflow and outflow pathway of the hippocampus and middle temporal lobe. The fornix is an arcuate fiber bundle from the hippocampus to the mammillary body. This tract provides a direct source of input from the hippocampal structure to the anterior nucleus of the thalamus and can effectively encode and integrate memory information (Browning et al., 2010; Lovblad et al., 2014). When the fornix of humans and animals is damaged, it can cause severe memory impairment (Tsivilis et al., 2008; Browning et al., 2010). Atrophy of the fornix can be accompanied by a transition from mild cognitive impairment to AD (Copenhaver et al., 2006). Hamani et al. reported for the first time that fornix stimulation can improve memory, although only one patient in their study received DBS to treat obesity (Hamani et al., 2008). Since then, DBS in the fornix has gradually been applied to AD. Chronic fornix DBS can stabilize or slow memory decline and increase hippocampal volume and cerebral metabolism in some patients with AD (Laxton et al., 2010; Smith et al., 2012; Fontaine et al., 2013; Sankar et al., 2015; Lozano et al., 2016) and improve spatial learning memory and recognition memory and reduce amyloidosis, the inflammatory response, the loss of neurons and the local changes in brain volume in AD model mice (Hescham et al., 2013b; Zhang et al., 2015; Gallino et al., 2019; Leplus et al., 2019). Therefore, the fornix is a good target choice for the treatment of DBS in human clinical research and animal research on AD (Senova et al., 2020).

#### MT

In the Papez circuit, information related to memory is transmitted to the mammillary body through the fornix, and then to the ANT through the MT. Although the MT participates in the Papez circuit, MT DBS did not cause memory changes in patients with refractory epilepsy (Duprez et al., 2005). No beneficial memory effect of MT DBS was found in SD rats injected scopolamine (Hescham et al., 2015). Therefore, it is worth exploring whether DBS in the MT has a positive effect.

#### ANT

The ANT is part of the Papez circuit, receiving information from the mammillary body via the MT and projecting it to the cingulate gyrus. The functional interaction between the ANT and hippocampus is crucial for spatial memory and conditional learning (Dumont et al., 2010). In AD model rats, ANT DBS improves impaired spatial memory (Chen et al., 2014). This is consistent with the research by Zhang et al., even though ANT DBS did not improve the recognition memory of AD model rats (Zhang et al., 2015). ANT DBS appears to be a potential treatment for AD cognitive dysfunction. However, Hescham et al. did not seem to find any beneficial memory effects of ANT DBS in SD rats injected scopolamine (Hescham et al., 2015). In addition, at high current densities, ANT DBS disrupts the acquisition of contextual fear conditions in healthy rats and impairs the performance of rats in spatially alternating tasks. In this case, ANT DBS caused a functional depolarization block near the stimulation electrode, which greatly reduced the spontaneous discharge of the local neuron population. Extracellular recordings showed that under high current ANT DBS, the discharge rate of DG cells was reduced and hippocampal activity was suppressed (Hamani et al., 2010). The differences in the results of various animal studies may have multiple causes, and further research is needed to explore the impact of ANT DBS in AD.

#### EC

The EC is located in the anterior part of the parahippocampal gyrus and is a key area for information transmission to and from the hippocampus, forming a three-synapse circuit with the hippocampus. At the same time, EC is the first area affected in AD and is closely related to memory formation (Braak and Braak, 1991; Khan et al., 2014). EC DBS can enhance the spatial memory of wild-type mice, and promote the rapid proliferation of neurons in the dentate gyrus (DG) (Stone et al., 2011). Acute EC DBS can improve memory deficits induced by scopolamine, and increase the expression of c-Fos in the CA3 region (Hescham et al., 2015). Research by Zhang et al. showed that chronic EC DBS contributes to spatial memory and recognition memory deficits induced by Aβ_40_ (Zhang et al., 2015), which is consistent with research on AD transgenic mice (Xia et al., 2017; Mann et al., 2018). In addition, chronic EC DBS can significantly reduce Aβ and tau in the hippocampus of AD transgenic mouse models, as well as reduce total tau and phosphorylated tau in the cortical region (Mann et al., 2018). In another study, it was been demonstrated that EC DBS can increase synaptic activity by increasing synaptophysin levels, and promote low sedimentation clearance of tau through the lysosomal pathway, thereby exerting a beneficial effect on AD (Akwa et al., 2018).

#### CA1

The hippocampus is a brain area that is closely related to learning and memory functions, and is almost the central structure of memory-related circuits in the brain. The first region of the hippocampal circuit is the CA1, which mainly projects to the EC and subiculum and is very important for spatial memory (Igarashi et al., 2014). Acute CA1 DBS can improve memory deficits induced by scopolamine, and the beneficial effect of CA1 DBS is accompanied by increased expression of Fos in the cingulate gyrus (Hescham et al., 2015). No other studies related to DBS in AD have targeted the CA1 area to date.

Overall, the fornix, NBM, and EC are the preferred targets for DBS treatment in AD, even if there are other alternative targets. Currently, there are only two comparative studies of different stimulation targets for DBS in AD. Hescham et al. compared the effects of DBS in the ANT, CA1, MT, and EC in AD rat models induced by scopolamine (Hescham et al., 2015). CA1 DBS and EC DBS could improve memory deficits caused by scopolamine, and no beneficial memory effect was observed in the ANT or MT. A study had even shown that DBS in the hippocampus and entorhinal regions can impair memory (Jacobs et al., 2016). Zhang et al. investigated the effects of DBS in the ANT, EC, and fornix on the cognitive behavior of AD rat models (Zhang et al., 2015). DBS of these three targets can benefit the spatial memory of AD model rats. EC DBS and fornix DBS also improved the recognition and memory of AD model rats, but this effect was not observed in ANT DBS. In the Papez circuit, the EC and the fornix are directly connected to the hippocampus, while the ANT is connected to the fornix and the nipple body and indirectly connected to the hippocampus. This difference in neural connectivity may explain why the EC and fornix show more significant spatial and recognition memory improvements than the ANT. The comparison of different targets of DBS in AD still needs more research.

### Stimulation Frequency

To date, the application of DBS in AD has been carried out under fixed stimulation parameters with a single frequency setting of 20, 100, or 130 Hz. The selection of frequency parameters in AD is based on the application of DBS in other diseases. The stimulation frequency of DBS, it can be divided into high frequency electrical stimulation (HFS, 25~1,000 Hz) and low frequency electrical stimulation (LFS, 0.1~25 Hz) (Schiller and Bankirer, 2007). HFS is commonly used in the treatment of mental illness. For patients with Parkinson's disease, high-frequency stimulation is beneficial for dyskinesias, and low-frequency stimulation may improve some axial movement symptoms (Baizabal-Carvallo and Alonso-Juarez, 2016). However, stimulation at a frequency that is too high can cause functional lesions (Jakobs et al., 2019). Therefore, it is critical to optimize the stimulation frequency of DBS to minimize the side effects of electrical stimulation caused by the stimulation frequency. Huang et al. implemented four DBS frequencies of 10, 50, 100, and 130 Hz in the NBM of Aβ precursor protein/Presenilin1 (APP/PS1) mice and tested their spatial memory using the Morris water maze (MWM) (Huang et al., 2019). It was found that 10 Hz DBS had no effect. The latency during the learning period was significantly reduced by DBS at frequencies at 50, 100, and 130 Hz, and the number of passes and occupation time of the target quadrant and platform area in the exploration task increased significantly. Higher frequency (100 Hz, 130 Hz) stimulation was better than lower frequency (10 Hz, 50 Hz) stimulation, and a shorter latency, a larger number of times and a longer occupation time were observed at 100 Hz. Therefore, the optimal DBS frequency remains unclear. Some studies have shown that the efficacy of DBS in AD is not affected by frequency, as 10 Hz and 100 Hz show the same effect (Hescham et al., 2013b).

### Stimulation Start Time

The stimulation start time of DBS in AD is also closely related to the effect of treatment. The time from pathological appearance to clinical symptoms of AD may be as long as 20 years or more (Bateman et al., 2012; Villemagne et al., 2013; Selkoe and Hardy, 2016). It is one of the new interests in the treatment of AD to take measures to intervene the development of AD before clinical symptoms appear, and to delay or even prevent brain lesions (Alzheimer's Association, 2018; Fan and Wang, 2020). Several studies have shown that DBS intervention in the early stages of AD produces better results. Huang et al. administered NBM DBS to APP/PS1 mice at 4, 6, 9, and 12 months of age and performed MWM tests at the end of 13 months of age (Huang et al., 2019). APP/PS1 mice at 4–6 months of age are in the early stages of AD. The results showed that the escape latency of AD mice receiving DBS at 4 and 6 months of age was significantly reduced, and the occupation time of the target quadrant and the number of passes through the platform area increased significantly. DBS at 4 months of age produced the best results. However, DBS had little effect at 9 and 12 months of age. EC DBS was performed in TgCRND8 mice at 6 weeks and 6 months of age, but a reduction in amyloid plaque was found only in mice at 6 weeks. In clinical studies, Hardenacke et al. evaluated the effects of DBS on 8 AD patients and proposed that NBM DBS in the early stage of the disease or at a younger age may have a beneficial effect on disease progression (Hardenacke et al., 2016). NBM DBS can increase blood flow by more than 50% in the cerebral cortex and nerve growth factor (NGF) expression in the parietal cortex by ~68% in healthy rats at 4–6 months of age, but in aged rats at 29–31 months of age, blood flow increased by only ~25%, with no significant change in NGF in the parietal cortex (Hotta et al., 2009). This again proves that providing DBS at a younger age has a more favorable impact. Unfortunately, in a phase II clinical study, it was suggested that fornix DBS may benefit patients with AD who are ≥65 years of age, and that patients under 65 years of age may show worsening condition (Lozano et al., 2016). This has an effect that is opposite to the impact of NBM DBS in interventions for young AD patients (Hardenacke et al., 2016). The reason for this difference may be that young patients are not in the early stages of AD, or fornix DBS alone may not be suitable for the early treatment of AD, or among other possible reasons.

### Stimulation Duration

After DBS, AD patients' disease progression slowed significantly. This may be related to the stimulation duration of DBS, and the persistence of the effect is unclear. Animal research has shown that both acute and chronic DBS can cause long-term remodeling of the mouse brain. One hour of fornix DBS improved spatial memory deficits and caused local volume differences in various regions of the brain in AD mice. These changes can last at least 45 days, suggesting that the role of DBS in AD is more than immediate (Gallino et al., 2019). The improvement of spatial memory and recognition memory caused by DBS for 24 h in the AD rat model induced by Aβ_42_ can last for at least 4 weeks (Zhang et al., 2015). Chronic DBS (7 h/d) was performed on Toronto triple-transgenic (3 × Tg) AD model mice for 25 d, and the beneficial effects of DBS on recognition and memory in AD mice lasted at least 1 month. The after effects of DBS suggest that chronic DBS can also cause long-term changes in brain function (Mann et al., 2018). Huang et al. performed four durations of DBS (7, 14, 21, and 28 days for 1 h/days) in APP/PS1 mice at 4 months of age and performed the MWM and Aβ detection 30 d after the end of DBS (Huang et al., 2019). However, 7 d of DBS had almost no impact. DBS for 14, 21, and 28 days all improved the spatial memory of APP/PS1 mice and significantly reduced the soluble Aβ_40_ and Aβ_42_ levels in the hippocampus and cortex. DBS achieved the best results after 21 consecutive days of stimulation. This shows that the therapeutic effect of DBS is not directly proportional to the duration of treatment.

### Unilateral/Bilateral Treatment

Of the human clinical studies and animal studies of DBS in AD, only 5 studies used unilateral DBS (Turnbull et al., 1985; Lee et al., 2016; Huang et al., 2019; Koulousakis et al., 2020; Tsai et al., 2020). Although researchers believe that bilateral DBS seems safe for AD, there have still been a small number of adverse events (Ponce et al., 2016; Leoutsakos et al., 2018). Preliminary experiments by Huang et al. showed that bilateral DBS led to more severe complications and higher mortality (Huang et al., 2019). In fact, unilateral DBS can also improve the symptoms associated with AD and shows good neuroprotective effects and reversible side effects (Lee et al., 2016; Huang et al., 2019; Tsai et al., 2020). To improve the safety of DBS for AD, whether to switch to unilateral DBS in the future is worth exploring.

### Current Intensity

The efficacy of DBS in AD is affected by current density. Decreasing DBS current intensities of 200, 100, and 50 μA were applied to rats receiving scopolamine to study the effects of higher, medium, and lower current densities. At low currents, there was no significant difference in the time ratio of discrimination between the displaced and familiar objects in rats receiving DBS compared with the control rats. However, the spatial memory of rats was substantially improved under DBS at 100 and 200 μA. Therefore, it was found that a lower current intensity had no effect on DBS in AD (Hescham et al., 2013b). However, there is no other study on the effect of DBS stimulation current intensity on AD.

## Potential Mechanisms of Action

The mechanism of DBS in AD is unknown. This article will explore the potential mechanism of DBS in AD from several perspectives.

### Regulation Related Neural Networks

**Figure 3:**
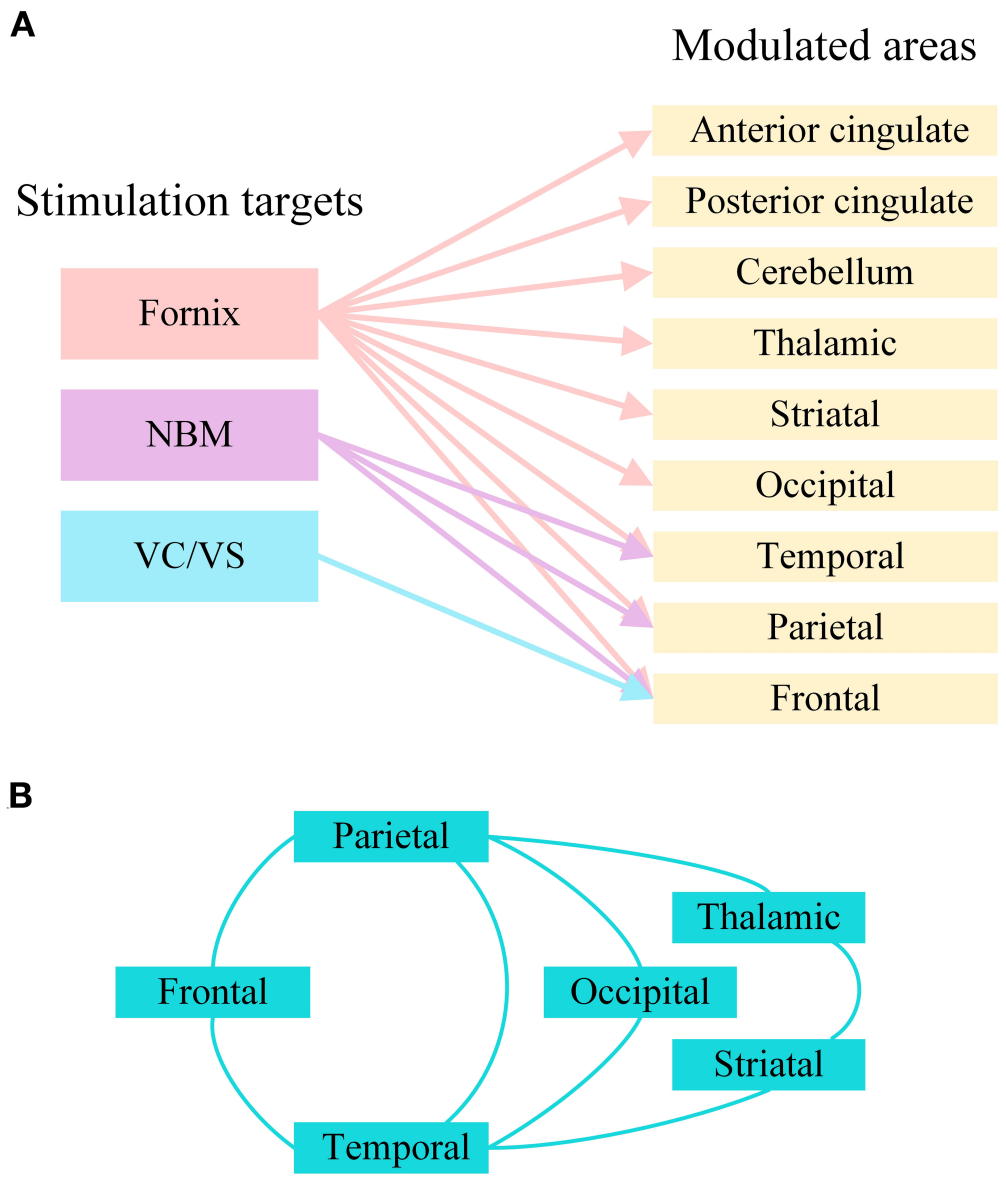
The modulated brain structure of AD patients after DBS in the current study. **(A)** Modulated brain areas of AD patients after DBS of different targets. **(B)** Modulated brain networks of AD patients after DBS. NBM, nucleus basalis of Meynert; VC/VS, ventral capsule/ventral striatum.

Due to related molecular and structural abnormalities, the memory network of patients with AD changes (Sperling et al., 2010). There is evidence that the Papez circuit and the default mode network of AD patients are impaired and that the inherent connectivity in the default network is disrupted during resting states and cognitive tasks (Raichle et al., 2001; Greicius et al., 2004; Sperling et al., 2009). DBS has been shown to play a role in the regulation of neural networks in diseases. In patients with epilepsy, fornix DBS shows an electrical effect in the upstream hippocampus (Lozano and Lipsman, 2013; Miller et al., 2015). Hamani et al. used fornix DBS in obese patients, and electroencephalogram (EEG) showed that electrical activity in the hippocampus and parahippocampus was activated during stimulation (Hamani et al., 2008). Based on this, the Hamani team further used fornix DBS in AD, and the results showed that the neural network of the default pattern network and memory circuits in AD patients' brains were activated, including the entorhinal and hippocampal regions, and the connectivity between neural networks in the brain was also increased (Laxton et al., 2010). Functional connectivity analyses revealed that cerebral glucose metabolism in AD patients increased in a frontal-temporal-parietal-striatal-thalamic network and a frontal-temporal-parietal-occipital network after fornix DBS (Smith et al., 2012). The beneficial effects of NBM DBS in AD patients are significantly correlated with the fronto-parieto-temporal pattern of cortical thickness (Baldermann et al., 2018). Figure 3 shows the modulated brain structure of AD patients after DBS in the current study. Therefore, DBS may establish upstream and downstream effects in related neural network circuits by targeting key nodes of the neural network in the AD brain, increasing connectivity between networks, and thereby improving AD symptoms.

### Promotion of Nerve Oscillation

Neuronal oscillations are essential for information processing and communication between different brain structures. DBS has the potential to reset the unstable mode of neuron oscillations in AD, especially θ oscillations in the hippocampus (Hardenacke et al., 2013; Senova et al., 2018). With an approximately sinusoidal (4–10 Hz) EEG activity, θ oscillations can be recorded in edge circuits, are related to various cognitive processes, and play an important role in learning and memory (Buzsaki, 2002; Laxton et al., 2010). Various drugs that destroy cognitive functions reduce or eliminate hippocampal θ oscillations (McNaughton et al., 2007; Scott et al., 2012). The disruption of θ activity leads to impaired spatial and recognition memory, and the restoration of θ rhythm improves the learning ability of rats (Howlett et al., 2004; Hasselmo, 2006; Villette et al., 2010). Suthana et al. found that DBS caused the hippocampal θ rhythm to reset, optimally encode input information, and improve memory function in animal models (Suthana et al., 2012). In addition, electrical stimulation of perforated pathways in rodents can trigger θ phase reset, which creates favorable conditions for long-term memory enhancement (McCartney et al., 2004). Abnormalities in θ rhythm have been shown in AD patients and mouse models of AD (Klimesch, 1999; Scott et al., 2012). Based on these studies, we hypothesized that DBS may improve AD symptoms through neural oscillations that cause θ reset.

### Reduction of Aβ Levels

Aβ oligomers are one of the main neuropathological signs of AD. Aβ oligomers are highly neurotoxic, which may cause loss of synapses and neuronal damage (Hardy and Higgins, 1992; Perl, 2010), and affect circuit connectivity and network activities (Canter et al., 2016). Over the past decades, genetic, biochemical, and pathological evidence has revealed the importance of Aβ as a neuropathological marker of AD (Sperling et al., 2011; Barage and Sonawane, 2015). Acute DBS significantly reduced Aβ plaques in the hippocampus and cortex of 6-week-old TgCRND8 mice (Xia et al., 2017). Chronic DBS can also reduce Aβ and APP levels in 3xTg mice. The main source of Aβ production is the hydrolysis of APP. APP is hydrolyzed by β-secretase and γ-secretase to generate Aβ amyloid production pathways and non-amyloid production pathways are cleaved by α-secretase and γ-secretase (Barage and Sonawane, 2015; Chen et al., 2017; Kowalski and Mulak, 2019). Arrieta-Cruz et al. showed that high-frequency DBS can increase α-secretase activity in the hippocampus of TgCRND8 AD mice by a factor of two, which significantly increases synaptic plasticity in the CA1 region but does not change β-secretase activity (Arrieta-Cruz et al., 2010). In addition, Aβ can be cleared by internalization into glial cells (Kim et al., 2018), and DBS can regulate glial cell activity (Vedam-Mai et al., 2016; Xia et al., 2017). Therefore, DBS can reduce Aβ levels and improve the pathological state of AD, possibly by reducing Aβ production or increasing Aβ clearance.

### Reduction of Tau Levels

Neurofibrillary tangles containing aggregates of hyperphosphorylated tau protein are also one of the neuropathological signs of AD. Tau with an abnormally high degree of phosphorylation forms toxic paired helical filaments, severely impairs synaptic function and causes cell death (Mohandas et al., 2009; Joel et al., 2015). Fornix DBS has no significant effect on hippocampal tau or phosphorylated tau in Wistar rats. However, EC DBS can reduce the total tau and Ser416-phosphorylated tau in the cortex and hippocampus of 3 × Tg AD mice and increase neurogenesis in the dentate gyrus (Mann et al., 2018). A study by Akwa et al. also showed that EC DBS can reduce tau phosphorylation and accumulation of tau oligomers in the CA1 region of 3xTg AD mice, and increase tau autophagy-lysosomal degradation and synaptic protein expression (Akwa et al., 2018). Therefore, DBS may affect the degradation or clearance of tau in AD to reduce tau levels. However, the specific details are not yet clear.

### Reduction of Neuroinflammation

It has been recognized that neuroinflammation plays an important role in the development and progression of AD (Von Bernhardi, 2007; Le Page et al., 2017). The development of AD is closely related to the complex cascade that leads to the death of neurons. Normally functioning glial cells can express Aβ-related degradation enzymes or bind related proteins to promote the degradation and clearance of Aβ (Mulder et al., 2012; Yali et al., 2014; Kim et al., 2018). With the development of AD, glial cell malfunction can release excessive inflammatory factors and neurotoxic factors to produce neurotoxic effects, promote the cascade of Aβ and inflammation, and aggravate neuronal death and the progression of AD (Bagyinszky et al., 2017). In AD, astrocytes and microglia, the two main groups of cells driving neuroinflammation, exhibit high levels of abnormal activation (Cohen et al., 2013; Lopategui Cabezas et al., 2014). Chronic fornix DBS can reduce the degree of astrocytic and microglial reactivity and the extent of neuron loss in the cortex and hippocampus (Leplus et al., 2019). DBS activates astrocytes and microglia in the early stage after implantation; the degree of glial reactivity later decreases (Song et al., 2013). Whether DBS promotes the degradation and clearance of Aβ by activating additional protective glial cells, thereby reducing neurotoxicity and cascade reactions and ultimately downregulating glial cell levels and reducing neuroinflammation, is worth exploring.

### Regulation the Cholinergic System

Degeneration of the cholinergic circuit is a pathological manifestation of AD. AD patients and animal models have obvious cholinergic dysfunction involving abnormal ACh production and degradation, including choline acetyltransferase (ChAT) and acetylcholinesterase (AChE), respectively (Davies and Maloney, 1976; Perry et al., 1977; Schliebs and Arendt, 2011). Acetylcholinesterase inhibitors (donepezil, rivastigmine, galantamine) are approved by the U.S. Food and Drug Administration for the treatment of AD patients (Unzeta et al., 2016; Kaushik et al., 2018; Alzheimer's Association, 2020). High levels of ACh contribute to hippocampal θ oscillation and enhance memory (Verdier and Dykes, 2001; Micheau and Marighetto, 2011). Supplementation of exogenous ChAT can improve memory and cognitive dysfunction in AD model mice (Fu et al., 2004; Zhu et al., 2020). Studies have shown that DBS improves scopolamine-induced learning and memory deficits in rats (Hescham et al., 2013b, 2015). Scopolamine is a muscarinic acetylcholine receptor antagonist. In APP/PS1 mice, DBS reduced the level of AChE in the hippocampus and cortex while increasing the level of ChAT, which implies an increase in ACh (Huang et al., 2019). In rats with amygdala injury, DBS treatment can effectively compensate for amygdala injury and reduce the activity of AChE (Kadar et al., 2014). Therefore, DBS can regulate the cholinergic system, which may be one of the mechanisms by which DBS improves AD. However, a large-scale study on long-term use of donepezil hydrochloride in patients with AD showed that the donepezil hydrochloride cannot help AD patients after the first 6 months. Acetylcholinesterase inhibitors are not long-term effective in the treatment of AD (Arai et al., 2016, 2018). This prompts us to consider whether DBS improves AD directly or indirectly by regulating the cholinergic system, whether this regulatory improvement is long-term effective, and how long this improvement will last. At present, there is no relevant research to solve these problems. There are still many questions to be explored about the regulation of DBS on the cholinergic system in AD.

### Induction of NGF Synthesis

DBS may exert neuroprotective effects by inducing NGF synthesis in AD (Mashayekhi and Salehin, 2006; Hardenacke et al., 2013). NGF is the most typical neurotrophic peptide involved in regulating the survival and differentiation of neurons (Lindsay and Harmar, 1989; Sofroniew et al., 2001). NGF levels and metabolic pathways are clearly imbalanced in AD (Cuello et al., 2010). The decrease in NGF supply at the age-related basal forebrain cholinergic neuron cell level is similar to that observed in AD (Salehi et al., 2004). NGF gene therapy caused classic trophic responses in the brains of AD patients, including neuronal hypertrophy, axonal sprouting and activation of cell signaling (Tuszynski et al., 2015). The supply of NGF can provide long-term cholinergic nutritional support, thereby slowing or preventing cognitive decline in AD patients (Hardenacke et al., 2013). Studies have shown that unilateral NBM DBS can result in significantly higher NGF levels in healthy rats than observed before stimulation. Mecamylamine, the nicotinic blocker, can completely eliminate the secretion of NGF, which suggests that the projection of the basal forebrain may be the reason for the increase of NGF (Hotta et al., 2009). In AD disease, the ability of DBS to induce the release of NGF may be a pathway closely related to its mechanism, which also seems to be related to the cholinergic system of the basal forebrain.

### Other Potential Mechanisms

**Figure 4:**
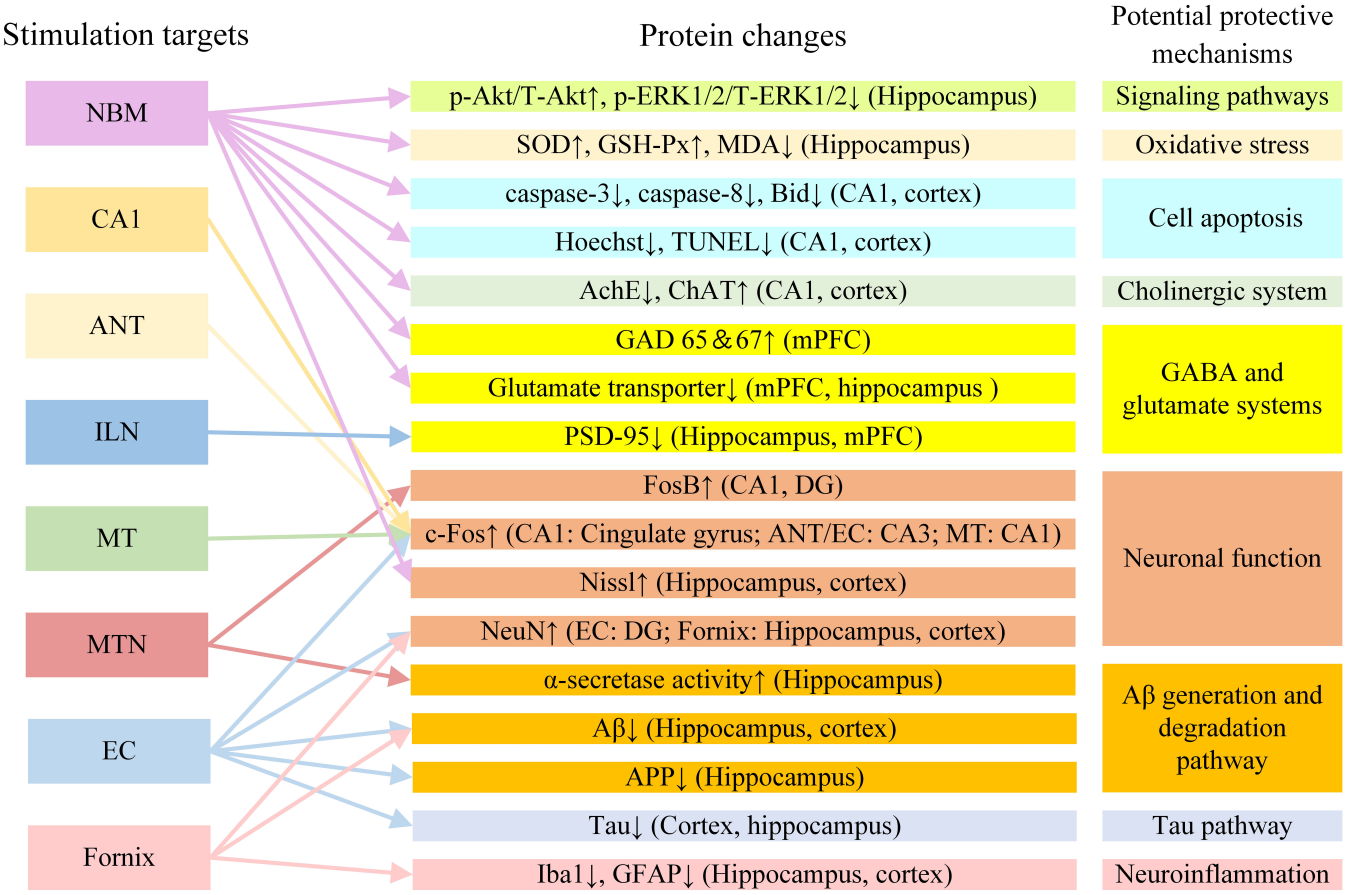
The protein changes and potential protective mechanisms in AD animal models after DBS in the current study. Aβ, β-amyloid; AchE, acetylcholine esterase; ANT, anterior nucleus of thalamus; APP, amyloid precursor protein; ChAT, choline acetyltransferase; EC, entorhinal cortex; DG, dentate gyrus; GABA, gamma-aminobutyric acid; GAD 65 and 67, glutamate acid decarboxylase 65 and 67; GFAP, glial fibrillary acidic protein; GSH-Px, glutathione peroxidase; ILN, intralaminar thalamic nucleus; MDA, methane dicarboxylic aldehyde; mPFC, medial prefrontal cortex; MT, mammillothalamic tract; MTN, midline thalamic nuclei; NBM, nucleus basalis of Meynert; PSD-95: postsynaptic density protein 95; SOD, superoxide dismutase; VC/VS, ventral capsule/ventral striatum.

The mechanism of DBS in AD may also involve a variety of other factors. In addition to the mechanisms described above, DBS may also improve AD symptoms by regulating other neurotransmitter systems. GABA and glutamic acid are closely involved in memory function (Sivilotti and Nistri, 1991). In AD rat models, glutamate acid decarboxylase 65 and 67 and glutamate transporter levels changed after NBM DBS. This suggests that NBM DBS may regulate changes in the GABA and glutamate systems and improve memory in AD rat models (Lee et al., 2016). It is also possible that DBS can enhance synaptic plasticity, promote neuron formation, and improve memory by regulating brain-derived nerve factors, including brain-derived neurotrophic factor and vascular endothelial growth factor (Gondard et al., 2015). It has been reported that DBS can increase hippocampal neurogenesis in AD (Mann et al., 2018; McKinnon et al., 2019). The newly born neurons have normal morphology and function and can promote the normalization of functional circuits (Lledo et al., 2006). DBS may improve AD symptoms by promoting neuron regeneration. Figure 4 shows the protein changes and potential protective mechanisms in AD animal models after DBS of different targets in the current study. The impact of DBS on AD animal models involves multiple potential protective mechanisms including signaling pathways, oxidative stress, cell apoptosis, the GABA and glutamate systems and neuronal function. More research is still needed to explore these possibilities.

## Discussion

This article reviews the published literature on DBS in AD. In patients and animal models with AD, DBS has shown some efficacy.

To provide the best parameter as a reference for the application of DBS in AD, this review summarizes and analyzes the different treatment parameters from current human clinical studies and animal studies of DBS in AD. Concerning stimulation targets, the NBM, fornix, and EC are the preferred targets for DBS treatment. This may be related to their structures and roles in the brain. They are involved in different circuit systems, including the base forebrain cholinergic system, the Papez circuit, and the trisynaptic circuit (Lv et al., 2018; Yu et al., 2019). There is currently no human clinical study of the EC as a target of stimulation in AD, and only animal studies support the EC as a target of DBS. The first area where neuropathy occurs in the AD brain is the EC, which then spreads to other cortexes and the hippocampus (Braak and Braak, 1991). The EC plays a very important role in information transmission. Whether EC DBS has a better effect than NBM and fornix DBS in AD is worthy of further investigation in animals and humans. In addition, EC DBS has been reported to improve spatial memory in patients with epilepsy by resetting the θ rhythm on EEG (Suthana et al., 2012).

Other DBS parameters that are commonly used in AD patient studies include stimulation frequency (130/20 Hz), stimulation duration (long-term), bilateral stimulation, pulse width (90–150 μs), and stimulation voltage (3.0–3.5 V) (Table 1); in AD animals the investigated parameters are stimulation frequency (130/100 Hz), stimulation duration (30 min-1 h, 3–42 days), bilateral stimulation, pulse width (60–100 μs), and stimulation current (100 μA) (Table 2). Based on the characteristics of human and animal tissues, although these DBS parameters used in AD animals cannot be directly applied for DBS in AD patients, they can provide more supporting evidence for the study of DBS in AD patients. Unilateral DBS should be popular in the treatment of DBS. After all, unilateral DBS is very likely to cause less surgical injury. Based on the long early incubation period of AD and the irreversible characteristics of neurodegenerative diseases (Selkoe and Hardy, 2016), using DBS to intervene in the early stages of AD to delay the AD process seems to be an excellent approach to treat AD (Huang et al., 2019). A number of studies have shown that NBM DBS and EC DBS have more beneficial effects in early AD (Hardenacke et al., 2016; Xia et al., 2017; Huang et al., 2019). However, in one clinical study, fornix DBS may have caused worse outcomes for AD patients at a younger age (Lozano et al., 2016). This may be related to multiple differences among different studies. In general, the preferred of DBS parameters for AD tend to be unilateral, early stage, and chronic treatment, with the NBS/fornix/EC as targets, and the pulse width, stimulation frequency, and stimulation voltage/current intensity can be individually designed in the future.

The improvement in AD caused by DBS is a multifactorial phenomenon, involving neural networks, θ oscillations, and changes in the microenvironment in the body (Laxton et al., 2010; Smith et al., 2012; Baldermann et al., 2018; Huang et al., 2019; Leplus et al., 2019). DBS is a neural regulation technology that directly changes brain activity in a controlled manner and corrects abnormal electrical circuits in the brain (Yu et al., 2019). In AD, during stimulation, DBS may have upstream and downstream effects on related neural network circuits by stimulating targets, activating or promoting electrical activity in the brain, and resetting θ oscillation. However, it is unclear what different upstream and downstream effects DBS has on different targets in AD. Most animal research focuses on the behavioral and biological effects in AD animals after DBS. The biological effects of DBS on AD animal models involve multiple potential protective mechanisms (Figure 4), and there may be interconnections among these fields. For example, DBS may regulate the microenvironment of AD animal models by promoting ACh release, inducing NGF synthesis, and reducing Aβ and tau levels (Hescham et al., 2013a; Xia et al., 2017; Huang et al., 2019). At present, these changes are only superficial phenomena, and the mechanism underlying them has not been explored. In the future, experimental methods such as electrophysiological recording, *in vivo* and *in vitro* optogenetics, and patch clamp technology may be used to further study the mechanism of DBS in AD.

DBS has broad application prospects for the treatment of AD. However, research in this area is still in its infancy. The stimulation parameters and mechanism of DBS in AD need to be further explored. There are still limitations to applying DBS in AD. To date, most of the published studies have been performed with small sample sizes, especially human clinical studies. Some patients with AD in human clinical studies also used acetylcholinesterase medication when receiving DBS (Table 1), which may have confounding effects on the results. Compared with other electrical stimulation techniques, DBS is an invasive technique with multiple risks, including the risks of major surgery, such as bleeding, infection and other side effects (Doshi, 2011; Ponce et al., 2016; Barrett, 2017). Creating a personalized DBS treatment plan for each AD patient is a way to reduce the risk of DBS. In addition, sex is considered to be a factor related to the sex risk for AD. AD has a higher prevalence in women (Mielke et al., 2014). Most experiments in animal research are dominated by male animals. Fornix DBS can significantly improve the performance of male mice in the water maze without affecting the performance of females (Gallino et al., 2019). Human clinical studies have not considered the sex of patients with AD. Therefore, whether DBS can eventually become an effective approach for AD is still not clear. More animal studies and human clinical studies on the application of DBS in AD are needed.

## Conclusion

Most current research shows that DBS is a promising intervention for the treatment of AD. However, future studies of DBS therapy for AD should consider additional aspects, including individual differences, based on the diversity of DBS parameters. The stimulation parameters need to be standardized, and the after effect and mechanism of action need to be further explored.

## Author Contributions

YL and XWu were involved in study design. YL, YS, XT, and XWa contributed in literature search and review. YL and YS drafted the manuscript. XT and XZ helped with discussion and analysis. YL, YS, and WL prepared pictures and tables. YL, XWu, BS, and WH reviewed the final version making the necessary changes. All authors read and approved the final manuscript.

## Conflict of Interest

The authors declare that the research was conducted in the absence of any commercial or financial relationships that could be construed as a potential conflict of interest.
